# Health Promotion for Students of Veterinary Medicine: A Preliminary Study on Active Microbreaks and Ergonomics Education

**DOI:** 10.3390/ani13101641

**Published:** 2023-05-15

**Authors:** Julia Andrea Grünwald, Theresia Franziska Licka

**Affiliations:** 1 Department for Companion Animals and Horses, University of Veterinary Medicine Vienna, 1210 Vienna, Austria; 2 Royal (Dick) School of Veterinary Studies, University of Edinburgh, Edinburgh EH8 9YL, UK

**Keywords:** students of veterinary medicine, human–animal interaction, well-being, health promotion, self-efficacy, musculoskeletal discomfort and pain, musculoskeletal disorders

## Abstract

**Simple Summary:**

A relevant number of veterinarians leave the profession within the first years after graduation. In a preliminary study, we investigated the effects of very short, active interventions, called microbreaks, in 36 students of veterinary medicine. Students were encouraged to integrate these microbreaks into their days over 6 weeks, within the total observational period of 12 weeks. Additionally, information on the ergonomics of a variety of veterinary tasks was given in weekly interactive talks with practicing clinicians and a physiotherapist. At the start, many veterinary students reported musculoskeletal discomfort in the neck and the lower back. All students were able to incorporate the microbreaks well during their study activities and found them relieving. After 12 weeks, the participating students reported fewer painful body regions, and that their self-efficacy in potentially painful, risky, or dangerous interactions with animals in a veterinary setting had increased. The feeling of control over dangerous situations increased with dogs but decreased with horses. Most students wanted to continue the microbreaks in the future and judged the topic of the present study to be relevant to their profession.

**Abstract:**

Occupational hazards, such as psychosocial stressors, physical injuries from human–animal interactions, and physically demanding work tasks, are common in the veterinary profession, and musculoskeletal discomfort and pain (MDP) may already be present in veterinary undergraduates. This preliminary study investigates the effects of very short, active interventions, called microbreaks, in 36 veterinary students. At the start, participants had a high prevalence of MDP, especially in the neck and lower back. Within a 12-week observational period, 6 weeks of active intervention comprised teaching microbreaks (nine strengthening, stretching, and relaxation exercises; 30–90 s each) and a weekly veterinary-specific ergonomics education and discussion. After the intervention, participants reported fewer painful body regions and an increase in their self-efficacy in potentially painful, risky, or dangerous human–animal interactions. After the 12-week observational period, participants had increased self-efficacy in the maintenance of physical health and self-protection but decreased self-efficacy in healing injuries after veterinary human–animal interactions. Participants felt to have increased and decreased control over dangerous situations with dogs and horses, respectively, although self-efficacy in handling horses increased. Participants integrated microbreaks well into their undergraduate activities and rated the topic relevant to their (later) profession. This should encourage the inclusion of similar programs in undergraduate curricula.

## 1. Introduction

The veterinary profession is associated with demanding tasks, including manual handling of heavy loads, repetitive bending and twisting of the trunk, remaining in uncomfortable body postures over prolonged periods (e.g., during surgery or dental procedures), and resisting unpredictable movements of animals [1,2]. These are risk factors for the development of musculoskeletal discomfort and pain (MDP) that compromise the health of veterinarians in different fields of practice [1,2,3,4,5,6,7,8]. Veterinarians who perform awkward hand or grip movements have a high risk of suffering from MDP of the upper limb and spine [2,7,9]. In addition, age, the difficulty of work tasks, and the range of veterinary procedures carried out are risk factors for developing MDP, which are associated with a loss of productivity and sick leave [2]. Moreover, psychosocial factors, such as time pressure and lack of appreciation, are correlated with MDP in veterinarians [10,11], and work-related psychosocial stressors are recognized as accelerators for the onset of MDP [12]. There is overwhelming evidence of the high psychosocial challenges in the veterinary profession [13,14,15,16], including but not limited to dealing with animal suffering, euthanasia decisions, or animal slaughter and processing for food. Students of veterinary medicine (veterinary students) have some, if limited, exposure to these factors, but they experience additional academic stressors (e.g., long hours of revision and pressure to pass exams) [17,18,19,20]. The sum of these factors might already put undergraduate veterinary students at risk, especially as other activities of student life, such as sports or long periods on electronic devices, may also add to the overall risk of developing MDP. It may develop into chronic pain and disability in both students and veterinarians and is influenced by psychosocial factors, such as depression and maladaptive pain responses [21].

It seems essential for the veterinary profession to reduce the hazards of MDP because of physical and emotional workloads, but the best approach for prevention and recovery remains unknown, and studies show ambiguous results [22,23,24,25]. A combination of targeted measures that include workplace organization and modification, physical training tailored to the job, and workplace-specific ergonomics may promise some relief for veterinarians [5,7,26]. Moreover, education in young veterinarians and veterinary students should also focus on the development of personal resources, e.g., self-efficacy, as it can beneficially affect their well-being [27,28]. Veterinary students already have stressful lives; therefore, measures integrating into rather than adding onto study and practical work when gaining professional experience are needed.

The present intervention study investigates whether the effects of a physical exercise program in the form of microbreaks in combination with veterinary-specific ergonomics education have a measurable impact. Active microbreaks have been shown to be effective in enhancing physical and mental well-being among office workers [29]. Moreover, ergonomics training combined with intra-operative microbreaks has been documented to alleviate MDP in surgeons [30]. We documented the well-being of veterinary students, self-reported MDP, the feeling of being in control in potentially dangerous situations with animals, and the perception of one’s personal ability to cope with veterinary-specific situations. Hence, the main research objective was to assess whether a microbreaks program and ergonomics education reduce MDP in veterinary students and increase their self-efficacy and internal locus of control.

## 2. Materials and Methods

### 2.1. Recruitment of Veterinary Students

**Figure 1:**
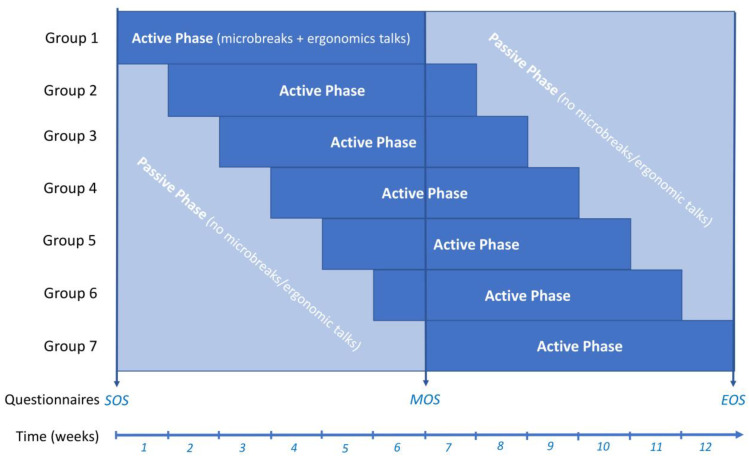
Timetable of the intervention study: Participants were able to choose from 7 groups with different starting dates of the Active Phase. They filled in the online questionnaires at the three time points in the week before the intervention started (SOS), in the middle of the study after 6 weeks (MOS), and after the intervention after 12 weeks (EOS).

Up to 60 veterinary students were invited to participate in the study. The study was advertised by posters at several locations on the Vetmeduni Vienna campus and social media platforms of the Students’ Union of Vetmeduni Vienna HVU. Students interested in participation could obtain further information online. After enrolment into the study via the digital teaching platform of Vetmeduni Vienna, students received an email with an informed consent form. After receipt of the signed consent form, each student was given an identification number for the pseudonymization of collected data. Within the study schedule, participants could choose from 7 groups with different starting dates of the Active Phase (Figure 1), with an online introduction to the Active Microbreaks Program with a physiotherapist (J.A.G.).

### 2.2. Data Collection Instrumentation (Questionnaires)

Questionnaires were created with the software tool SoSci Survey to provide online access. Participants filled in the online questionnaires at the three time points SOS, MOS, and EOS (Figure 1). These were sent to participants in the week before the intervention started (SOS), in the middle of the study after 6 weeks (MOS), and after the intervention after 12 weeks (EOS). We used identification numbers for data collection. Questionnaires included baseline demographics, questions regarding dealing with pain using analgesics and/or physical interventions, the prevalence of MDP, and questions regarding the participants’ self-efficacy and the feeling of being in control in veterinary-specific situations. Regarding pain management, participants were asked how they dealt with pain answering the items: “When I have pain, I take something for it (e.g., pain-relieving substances)” and “When I have pain, I do something about it (e.g., exercise, relaxation, physiotherapy)”. Answers were given on a 6-point Likert scale (“strongly disagree” (0) to “strongly agree” (5). To assess MDP, a modified German version of the Standardized Nordic Musculoskeletal Questionnaire [31] (NMQ), a frequently used questionnaire for symptoms of musculoskeletal disorders, and the German version of the STarT-Back Tool, validated to screen primary care patients with low back pain for prognostic indicators [32], were used. With a psychosocial subscale, the STarT-Back Tool divides participants into low, medium, or high-risk categories for persistent disabling back pain. The high-risk category is associated with depression, anxiety, and fear–avoidance beliefs [33]. In addition, a questionnaire to document external and internal locus of control (LOC), developed after Rotter [34], based on coping with difficult, i.e., potentially painful and/or dangerous human–animal interactions, was used. The locus of control reflects the perceived ability to be in control of a situation. It distinguishes an internal from an external locus of control, with the situation controlled by oneself or other people, animals, or circumstances, respectively. Furthermore, participants completed a questionnaire on self-reported general self-efficacy (GSE) and specific self-efficacy (SSE) for veterinary students developed after Bandura [35]. Self-efficacy reflects one’s perceived ability to perform tasks and activities efficiently. The items for veterinary students’ LOC, SSE, and GSE were developed for the present study by the authors. They included items that were considered relevant for veterinary students when entering veterinary work fields and facing veterinarian-specific stressors, e.g., potentially dangerous or stressful human–animal interactions. These items are based on references in order to assess differences in self-efficacy and locus of control in veterinary students´ specific environments. Items were rated on a 6-point Likert scale from “strongly disagree” (0) to “strongly agree” (5). At EOS, an additional questionnaire concerning relevance, relaxation, and human–animal interactions was filled in, and items were rated on a 6-point Likert scale from “strongly disagree” (0) to “strongly agree” (5). Additionally, the feasibility and impact of the microbreaks program on the participant’s life were rated on a dichotomous scale (“Yes” or “No”) or “Does not apply”, respectively.

### 2.3. Data Analysis

For data analyses, SPSS 28.0.1.0 statistical software and Excel 2019 software were used. To measure a time–dose effect, a bivariate correlation, Spearman’s Rho *r_s_*, was applied to the results of MOS and the weeks of the Active Phase. The Wilcoxon signed-rank test was applied to compare the results of SOS and EOS. The level of significance was set at *p* < 0.05.

### 2.4. Intervention

The study duration for each participant was 12 weeks, with an active participation phase of 6 consecutive weeks (Active Phase) and 6 weeks of non-active participation (Passive Phase). The decision for this staggered intake of participants over 7 weeks (Figure 1) was made to allow the assessment of a time–dose effect at MOS and other confounding factors, such as scheduled exam times or the development of the COVID-19 pandemic. This was also the reason for adapting the intervention from face-to-face to online. Furthermore, staggered intake allowed for the participation of students, e.g., on externships during some weeks.

### 2.5. The Active Phase Consisted of Two Parts

- Active Microbreaks Program: An Active Microbreaks Program, tailored to veterinary students, was offered via online sessions by a physiotherapist (J.A.G.). Microbreaks are frequent but short breaks to be done parallel to work-related activities. The Active Microbreaks Program consisted of 9 strengthening, stretching, and relaxation exercises and, hence, 9 active microbreaks. The introduction to the Active Microbreaks Program additionally included some strategies for body-friendly working postures. Via online video conferences, participants were instructed on how to perform the microbreaks and how to include them in their daily study and work routine. One active microbreak was expected to take between 30 and 90 s. Participants were encouraged to select microbreaks depending on their specific needs. After the introduction to the Active Microbreaks Program, participants were asked to incorporate the exercises into their student life on a daily basis for the following 6 weeks. Which exercises were chosen by participants, the number of repetitions of each microbreak, and the time spent performing the microbreaks were not controlled for, which is a limitation of the present study.
- Ergonomics talks: An optional weekly educational “ergonomics talk” with experts of clinical veterinary medicine from their specialized fields was offered additionally. The ergonomics talks had 11 topics, focusing on workplace challenges in equine dentistry, equine orthopedics, equine internal medicine, small animal practice, small animal surgery, small animal emergency, bovine medicine, small ruminant medicine, pig medicine, as well as workplace challenges when driving a veterinary practice van and working at a computer screen. Video content of work tasks specific to the 11 topics was discussed between the physiotherapist (J.A.G.) and one veterinary expert in that field. The aim was to highlight opportunities to optimize ergonomic working practices without compromising safety and efficiency. These ergonomics educational sessions were offered to underline the usefulness of including active microbreaks into the veterinary student´s daily study and work routine for their later careers. Students could participate live online or re-watch the session online for a week after its recording. Neither participation in ergonomics talks nor re-watching the sessions was controlled for.

## 3. Results

### 3.1. Baseline Demographics

Of the 47 students who were interested in the study, 38 signed their consent form and completed SOS. After 12 weeks of the study duration, two participants did not complete EOS and were therefore excluded from the data analyses. Of the 36 remaining participants (*n* = 36), the age range was 19 to 33 years, with a mean of 23.5 years (*SD* = 2.7) and a median of 23 years, with 33 participants indicating to be female and 3 to be male. Participants reported to be between the 4th and 12th semester of their study of veterinary medicine; more than half of the participants were in the 6th semester. Two participants were in the Small Animal Medicine Specialization, the Ruminant Medicine Specialization, and the Public Veterinary Care and Health Care Specialization. One participant was in the Conservation Medicine Specialization.

### 3.2. Strategies for Coping with Pain: “I Do Something” (Physical) Versus “I Take Something” (Medical)

**Figure 2:**
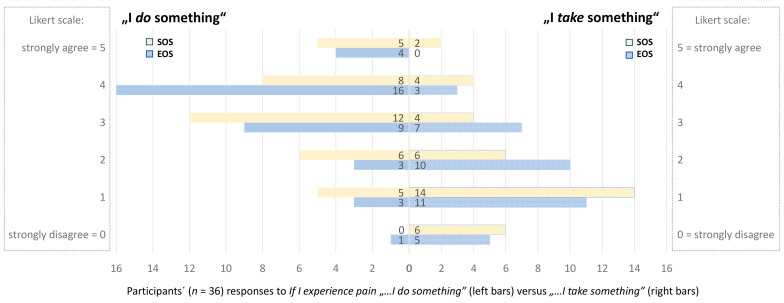
Coping with Pain Strategy: Responses to the items “If I experience pain, I DO something” (physical) versus “If I experience pain, I TAKE something” (medical) at the time points in the week before the intervention started (SOS) and after the intervention after 12 weeks (EOS) on a 6-point Likert scale from “strongly disagree” (0) to “strongly agree” (5). *n* = the total number of participants.

There was no significant difference between SOS and EOS answers; 15 participants indicated a non-significant increase in having done more/more often physical interventions against pain, and 9 participants who chose lower values indicated having done less/less often physical interventions at the end of the study compared to before the study. Comparing data from time points SOS and EOS, 9 participants indicated increased their use of pain-relieving substances, and 10 indicated the opposite. Details of these responses are in Figure 2. The comparison of SOS and EOS is in Table 1.

### 3.3. Nordic Musculoskeletal Questionnaire (NMQ)

**Figure 3:**
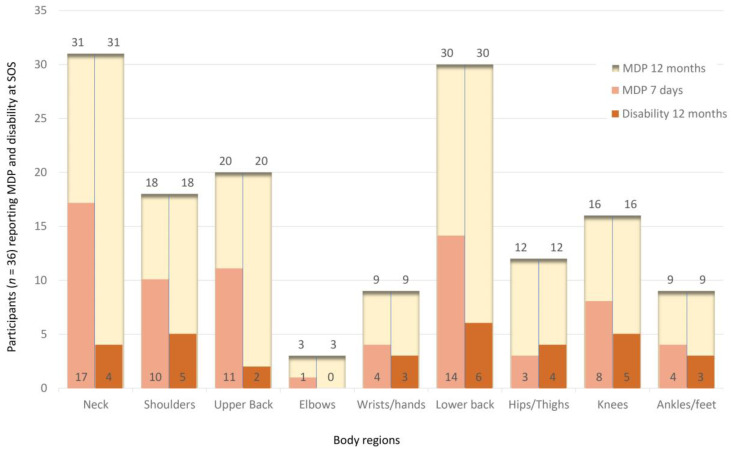
Agreement to the items in the Nordic Musculoskeletal Questionnaire reporting musculoskeletal discomfort and pain (MDP) at the time point in the week before the intervention started (SOS): The large upper bars in the background (MDP 12 months) reflect the responses to the question for each body region: “Have you at any time during the last 12 months had any discomfort or pain?” The right bars in the front reflect the responses to the question for each body region (Disability 12 months): “Have you at any time during the last 12 months been prevented from doing your normal work (at work, home, or leisure activities) because of the discomfort or pain?” The left bars in the front reflect the responses to the question for each body region (MDP 7 days): “Have you had any discomfort or pain at any time during the last 7 days?” *n* = the total number of participants.

**Figure 4:**
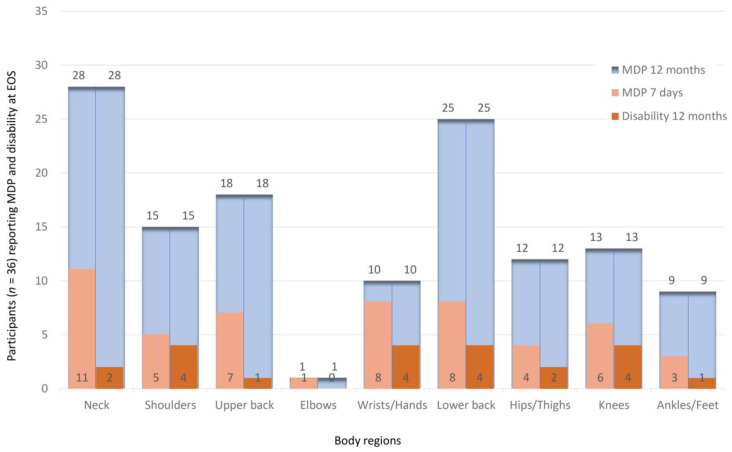
Agreement to the items in the Nordic Musculoskeletal Questionnaire reporting musculoskeletal discomfort and pain (MDP) at the time point in the week after the intervention after 12 weeks (EOS): The large upper bars in the background (MDP 12 months) reflect the responses to the question for each body region: “Have you at any time during the last 12 months had any discomfort or pain?” The right bars in the front reflect the responses to the question for each body region (Disability 12 months): “Have you at any time during the last 12 months been prevented from doing your normal work (at work, home, or leisure activities) because of the discomfort or pain?” The left bars in the front reflect the responses to the question for each body region (MDP 7 days): “Have you had any discomfort or pain at any time during the last 7 days?” *n* = the total number of participants.

The 12-month and 7-day prevalence of MDP, as well as the disability of normal activity in the preceding 12 months, is shown in Figure 3 at SOS and Figure 4 at EOS. The neck was the most prevalent painful body region reported by participants at SOS as well as at EOS. At SOS, as well as at EOS, 24 participants reported having had up to four painful body regions in the preceding 12 months, and one reported having had no painful body region. At SOS, 17 participants reported being kept from normal activities in the preceding 12 months due to musculoskeletal symptoms in up to four body regions, compared to 12 participants at EOS. At SOS, 26 participants reported having up to four painful body regions in the previous 7 days, whereas 22 reported up to four body regions as painful at EOS. Regarding the 7-day prevalence at SOS, only 7 participants reported having no MDP versus 13 participants at EOS. The sum of different body regions that each participant reported to be painful in the preceding week was significantly different between SOS and EOS, as documented by the Wilcoxon signed-rank test for pairwise comparisons (see also Table 1).

### 3.4. STarT-Back Tool

The results of the STarT-Back Tool (assigning low, medium, or high risk for persistent disabling back pain) showed no statistically significant change in scores at SOS and EOS when the Wilcoxon signed-rank test was used. At SOS, 32 participants (of 36) were at low risk for chronification of back pain, and at EOS, 33 participants (of 36). Two participants improved from medium risk at SOS to low risk at EOS, and one deteriorated from medium to high risk at EOS. One participant was at high risk at both SOS and EOS (see also Table 1).

### 3.5. Locus of Control (LOC)

On a 6-point Likert scale from “strongly disagree” (0) to “strongly agree” (5), participants were asked for their locus of control when dealing with veterinary-specific situations. The median values of the items for the external locus of control were between 1 and 3, while the median values of the items for the internal locus of control were between 2 and 5 (see also Table 2).

### 3.6. Specific Self-Efficacy (SSE)

Participants were asked for their specific self-efficacy when dealing with veterinary student-specific situations on a 6-point Likert scale from “strongly disagree” (0) to “strongly agree” (5). Three of the six items showed a significant increase in scores regarding self-efficacy after the intervention. The items “I know what to do in situations that are often painful for me (during my work with animals)” and “I know how to behave in situations with animals that are probably dangerous (during my work)” showed a strong effect and the item “I know how to prevent risky situations with animals (during my work)” showed a medium effect. Hence, the Wilcoxon signed-rank test revealed a significant effect on participants´ self-reported assessment, with the lowest values at SOS and the highest values at EOS (see also Table 2).

### 3.7. General Self-Efficacy (GSE)

On a 6-point Likert scale from “strongly disagree” (0) to “strongly agree” (5), participants rated their general self-efficacy when dealing with veterinary-specific situations. Three of five items showed a significant increase in scores with a medium effect at EOS. The Wilcoxon signed-rank test revealed that the scores of the items “I am confident that I know how to protect myself” and “I am confident that I can maintain my physical well-being” were significantly higher at EOS. Results indicated that after the intervention at EOS, participants felt more confident handling horses, but not dogs or cats (see also Table 2).

### 3.8. Time-Dose Effect of Weeks of Active Phase

For the time-dose effect at MOS, five correlations were significant with a medium effect. Three of the significant results regarded the prevalence of MDP in the preceding 12 months within the NMQ and one significant result each within the LOC and the SSE (see Table 3): Participants’ self-assessment of their MDP correlated significantly with the weeks of the Active Phase, r_s_ = 0.307, *p* = 0.034, *n* = 36. A time–dose-effect analysis revealed that more participants reported feeling pain in the knee and hip in the preceding 12 months, with the number of weeks of the Active Phase. They also reported more body regions being painful with longer participation in the study. The items “If I take the right actions, I can avoid being kicked by a horse” and “In the veterinary profession, it is possible to be injured by animals while working (human–animal interaction): I am nevertheless confident of recovering in my career as a veterinarian” showed a negative correlation with the number of weeks of active participation.

### 3.9. Questionnaire on Relevance, Relaxation, and Human–Animal Interactions at EOS

**Figure 5:**
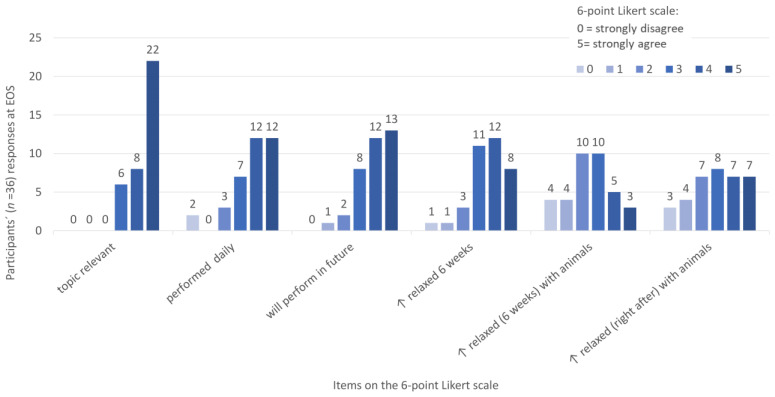
Responses to the items concerning relevance, relaxation, and human–animal interactions at the time point in the week after the intervention after 12 weeks (EOS) on a 6-point Likert scale from “strongly disagree” (0) to “strongly agree” (5): The long version of the abbreviated items in the figure from left to right are: “The topic of the study: ‘Health promotion for students of the Vetmeduni Vienna with a short microbreaks exercise program’ is relevant for my profession.”; “I have performed the microbreaks/some of the microbreaks every day.”, “I will continue to perform the microbreaks/some of the microbreaks in the future.”, “I feel more comfortable/relaxed after 6 weeks of performing microbreaks than before.”, “After 6 weeks of performing microbreaks, I feel more comfortable handling animals.”, “I feel more comfortable handling animals right after I have performed the microbreaks”. *n* = the total number of participants.

After the intervention at EOS, participants responded to a questionnaire on a 6-point Likert scale from “strongly disagree” (0) to “strongly agree” (5). The results are depicted in Figure 5.

### 3.10. Questionnaire on Feasibility of Microbreaks for Veterinary Students at EOS

All participants who completed the study reported that implementing the microbreaks program worked well during their study activities. Further, 11 participants agreed with the item “I was able to implement the microbreaks program well at university”. For 11 participants, this item did not apply because they were not at university (probably due to online courses, externships, or because of lockdown regulations) during their Active Phase. For 13 participants, the item “I was able to implement the workout program well during my (part-time) job” applied. For 16 participants, this item did not apply, indicating that they had no (part-time) job at that time. Seventeen study participants agreed with the item that they were able to implement the microbreaks program well while handling animals. Ten participants did not agree. This item did not apply to nine participants, indicating that they had no direct contact with live animals during their Active Phase.

### 3.11. Relief and Positive Impact of the Microbreaks at EOS

All participants who finished the study stated that the microbreaks were a relief from their study activities load, with 13 (of 36) reporting that the microbreaks were helpful at university and 11 disagreeing. Eighteen participants reported a positive impact on their life at university, and six did not agree. Twelve participants indicated they had not been at university during their Active Phase. Microbreaks had a positive impact on study activities for 33 (of 36) participants, and one student reported no positive influence on their study activity. In contrast, two participants stated, “does not apply”, indicating they had not had study activities during their Active Phase. Sixteen participants reported relief at their (part-time) job and three disagreed. Microbreaks had a positive influence on the (part-time) job(s) for 17 participants, and two participants disagreed. Seventeen participants indicated not having worked during their Active Phase. For 21 participants, the exercises were helpful when handling animals, and six participants disagreed. A positive impact on handling animals was reported by 22 participants, while five disagreed. Nine participants indicated they had had no contact with live animals during their Active Phase.

## 4. Discussion

### 4.1. Limitations with COVID-19

The present study was conducted during the COVID-19 pandemic; hence, results may be biased by practical changes and other impacts on veterinary students [36,37,38,39]. Furthermore, COVID-19 increased sedentary behavior in Swiss students of various courses [40], and remote or e-learning could have accelerated the prevalence of MDP [41,42,43]. When MOS took place, a COVID-19 lockdown in Austria could have affected the prevalence of MDP, especially because increased health anxiety during COVID-19 was associated with increased reports of MDP, particularly in women [44]. In the present study, the age range of participants was 19 to 33 years, and the main gender was female, which is relevant as young adults and women showed the most deterioration of mental health during the COVID-19 pandemic and lockdowns in Austria [45]. Uncertainty, distress, and complicated grief during COVID-19 affected U.S. veterinary students [36]. A decrease in well-being and an increase in psychosocial work demands were found within veterinary academia [46] and among veterinary students [38]. This may also have been the case for participants in the present study. Physical activity decreased despite its positive impact on mental health during the COVID-19 pandemic [45], but there was an increase in exercising indoors with online instructions by about 63% in Austria [47]. Unlike our original plan, we used similar online sessions to introduce the microbreaks program and the ergonomics talks. The physical activity of the microbreaks program could have buffered COVID-19-related stressors, but this was not investigated in the present study.

### 4.2. Musculoskeletal Discomfort and Pain (MDP)

Veterinary work comprises physical [1] and psychological [15] challenges potentially detrimental to the well-being of professionals working with animals [48,49]. Among occupational hazards [50,51,52], MDP is a common health threat for veterinarians [1,2,3,5,7,8,9]. Compared to veterinarians overall [2,7,9], a higher annual prevalence of MDP in the neck was found in the participants of the present study, similar to veterinary surgeons during or after laparoscopic surgery [5]. For those veterinary surgeons, a microbreaks program similar to the one in the present study, including exercises and stretching supervised by physiotherapists and human doctors, was recommended but not investigated [5]. Interestingly, veterinary students were found to have higher muscular workloads of the upper extremity during simulated laparoscopy compared to simulated open laparotomy, which was thought to be because of the fine motor control challenges [26]. Compared to veterinarians in New Zealand [2], only the annual prevalence of MDP in shoulders, elbows, and wrists/hands was reported less by the participants of the present study. Veterinarians reported MDP in the lower back most frequently [2], and the participants of the present study reported this even more frequently. Veterinarians working as clinicians in Turkey reported that during their student days MDP had already occurred in the back (15.1%), shoulders (14.1%), neck (13.6%), arms (13.6), and lower back (13.6%) [10]. Similar to the present study, elbows were the least reported [10]. Compared to the results of the present study, these values are much lower, possibly due to a recall bias, as the veterinarians’ self-reports were given well after graduation. We suggest that MDP present in veterinary students, as found in the present study, may develop into the high prevalence of MDP in veterinarians documented in many studies, but this remains to be investigated in future longitudinal studies.

### 4.3. Microbreaks and MDP

At EOS, a decrease in body regions experiencing MDP in the preceding 7 days was found, similar to the decrease in MDP experienced by human medical surgeons performing microbreaks during surgery, especially in the neck, wrists, shoulders, and lower and upper back. In that study, microbreaks also enhanced physical performance and mental focus without prolonging surgery time despite microbreaks of 90–120 s [53]. Similar to the present study with 36 undergraduate veterinary students, 15 surgical residents [54] and 8 gastroenterologist fellows [55] were offered ergonomics education and a microbreaks program without significant reductions in pain or discomfort. Wrists and hands were not specifically featured in the present study, but as a 6-week exercise program to increase hand grip strength for veterinary students improved the accuracy of bovine pregnancy diagnosis [56], future intervention programs should include stretching, strengthening, and relaxation exercises for hands and wrists. Moreover, it has already been successful in a microbreaks intervention study for human surgeons [53].

### 4.4. Time–Dose Effect and MDP

With the duration of this study, body regions reported to have experienced MDP in the preceding 12 months increased significantly. With the duration of the Active Phase, the 12-month prevalence of MDP in hips and knees increased. Chronic pain and attentional focus can increase reporting of the frequency or intensity of pain [57], and it is possible that participants developed increased body awareness, including awareness of unpleasant sensations. This may lead to increased reports of pain and discomfort, significantly, as pain perception is altered by stress or anxiety [58], and veterinary students have been shown to experience psychological distress [17,19].

### 4.5. Psychosocial Stress and MDP

Occupational stress and psychological issues are known to have a longitudinal effect on the development of musculoskeletal discomfort or pain [59,60]. In addition, psychosocial risk factors for the development of MDP in veterinarians have been documented in various studies, namely high job stress and low job satisfaction [10] or personal and workplace issues [11]. Furthermore, animal-induced accidents are a major risk for veterinarians [48,61,62,63,64,65,66,67,68,69]. The anxiety of being seriously injured could enhance the development of MDP, as anxiety is associated with the prevalence of MDP [70]. This assumption should be tested in future studies regarding anxiety about human–animal-related incidents and the prevalence of MDP.

### 4.6. Coping with Pain Strategy

With the microbreaks program in the present study, the authors aimed to add an adaptive coping strategy for dealing with bodily discomfort or pain. Research has identified self-medication, regular analgesics use in veterinarians [3,5,49,71], and legal substance use in veterinary students [72]. Among bovine practitioners experiencing MDP, over-the-counter medication was used by over 75%, followed by physical interventions, e.g., physiotherapy, massage, and exercise therapy, with about 40% each, and prescription medication over 25% [8]. This is in contrast to the present study, with about three-quarters of participants (indicated by agreements with values 3–5) using a physical intervention strategy, e.g., exercise, relaxation, physiotherapy, and more than one-quarter of participants (indicated by agreements with values 3–5) using pain-relieving substances when experiencing pain. In another study, female veterinarians more regularly used medications, and intake particularly increased for veterinarians under psychosocial stress and demoralization [71]. In addition, the consumption of psychotropic substances was used as a coping strategy for psychosocial stress [71]. A proportion of veterinary students lack adaptive coping strategies [20,73] and use unhealthy drinking behavior to manage negative emotions [74]. Among German and Austrian female veterinarians, maladaptive coping strategies, e.g., rumination or resignation in stressful occupational situations, were also found. Moreover, veterinarians were less likely to be able to relax in general or in single body parts [75]. Adopting a problem-focused or an emotion-focused coping strategy, e.g., physical exercise or relaxation techniques, is recommended to manage stress in difficult or traumatic veterinary settings [76]. Stress management training is recommended for veterinary students and veterinarians [75]. In the same vein, researchers in the Netherlands proposed stress management interventions aimed at improving personal resources to promote well-being early in veterinary students [28]. In the present study, no significant changes in participants´ strategy when coping with pain were observed despite a substantial increase in self-efficacy among the item “I know what to do in situations that are often painful for me (during my work with animals)”. In addition to beneficial physical impacts, the present microbreaks program and ergonomics education could foster the development of personal resources in veterinary students and add a tool for improving self-efficacy when confronted with stressful or painful situations within their studies.

### 4.7. Gender and MDP

Female veterinary students especially perceive lower self-efficacy than male colleagues [28], and MDP was reported especially among women working in the veterinary field [5,6]. The main gender in the present study was female, and this is representative of veterinary students and the veterinary field at large (in the Western world). Therefore, elevated reporting of MDP could have also occurred due to gender bias. Work environments (e.g., tables, tools) may not be ideally suited to the mainly female participants in the present study population. Women surgeons of human medicine reported significantly more cases of MDP in the neck, upper back, and shoulders and treatment for hand-related injuries than their male colleagues [77], possibly due to inappropriate heights of operating room tables requiring raised arms, leading to MDP of the upper extremities in women [77]. Regarding the ongoing feminization of the profession of veterinary medicine [75,78], a central focus for the prevention of MDP should be set on the inclusion of gender aspects to allow the best possible measures in the occupational prevention of MDP in veterinary medicine. While the majority of students of veterinary medicine in Austria are female and, therefore, participants were representative; the findings may not be transferrable to male veterinary medicine students.

### 4.8. Locus of Control

In the present study, participants agreed significantly more frequently with the item “If I get bitten by a dog, it’s my own fault” at EOS compared to SOS. A recent study found that physical activity, as was included in the present study, might foster or even lead to regaining a sense of control and, thus, might promote mental health in students [79]. Efforts to increase one’s sense of control could be a promising strategy, including in the prevention of burnout [80]. Hence, interventions involving strategies to facilitate a sense of control could be especially beneficial for veterinary students and young veterinary professionals who already face study-related psychological issues, e.g., compassion fatigue or burnout [17,19,81]. Contrary to the above-mentioned “Dog bite” item, at MOS, there was a negative time–dose effect and, therefore, less agreement with the item “If I take the right action, I can avoid being kicked by a horse”. The longer participants were in the Active Phase of the study, the lower their confidence in this regard. One reason for this could be that participants were exposed to information about dangerous situations with horses in the ergonomics talks. The horse-related injury rate is much higher than the dog-related injury rate [82], and injuries from horses can be serious or even fatal. Despite this, veterinary and animal science students were reluctant to comply with the safety policies, such as wearing protective boots, even though they also reported having experienced horse-related injuries [68]. For future interventions, veterinary students should be provided with effective practical advice for the prevention of injuries and accidents with horses (and other large animals) and with safety measures, e.g., sedation [66]. Improving the predictability of horses could not only advance safety in human-horse interactions [69,83,84] but also improve the feeling of being in control when veterinary students handle horses. Hence, it may work as an adaptive coping strategy for potentially dangerous human–animal interactions. The information on preventive measures and outline of negative consequences of risk-taking increases the internal locus of control in students taking part in workplace safety and health training [85]. This could be protective not only regarding accidents during human–animal interactions but could also enhance coping with other stressful situations. It must be noted that with acceptance of horse-related injuries and deaths in the equestrian culture [66,69,83,84], bravery and risk-taking in veterinarians, as well as the culture of working through pain, might also hinder safe occupational working styles [53]. While improvement in proper patient safety seems self-evident [86], the lack of adequate safety measures also leads to veterinarians having a high incidence of work-related accidents [48,61,62,63,64,65,66,67,68,69,87].

### 4.9. Self-Efficacy

The agreement with the statements “I know what to do in situations that are often painful for me (during my work with animals)”, “I know how to prevent risky situations with animals (during my work)”, and “I know how to behave in situations with animals that are likely to be dangerous (during my work)” increased significantly at EOS. Similarly, there was an increased agreement to the self-efficacy items “I am confident that I know how to protect myself” and “I am confident that I can maintain my physical well-being”. Participants at EOS were more confident in their knowledge of self-protection and maintaining their physical well-being in general, indicating that participants increased their self-efficacy. An increase in self-efficacy could have ameliorated pain or discomfort in the present study. Depending on the context and task, self-efficacy can be considered a protective factor for stress, pain [88], and pain development [89]. In an intervention study for veterinary students, the development of personal resources, e.g., self-efficacy, was found to support students´ well-being [27,28]. Self-efficacy was related to work engagement and was negatively related to cynicism in female veterinary students. Researchers advocated allowing students to develop important resources, e.g., reflective skills, a pro-active attitude, and self-efficacy [28]. Another study investigating veterinary students also found that self-efficacy and coping strategies influence the resilience of graduated veterinarians [90], reducing veterinary-specific occupational stressors. Documented by the time–dose effect and possibly associated with the LOC item “Horse kick”, participants became less confident about regaining their health after an animal-related injury (seen in a decreasing agreement to the item “In the profession of veterinary medicine, it is possible to be injured by animals while working (human–animal interaction): Given that, I am still confident of getting well again during my career as a veterinarian”). This may be a realistic perspective, as a recent study among 136 equine veterinarians showed that among 579 horse-related injuries in the preceding 5 years, 37% resulted in continued discomfort or loss of function [67]. This highlights the need for proper risk appreciation training [61] to enforce safety behaviors and reduce injuries in students and veterinarians. On the other hand, agreement to the item “I am confident in handling horses” significantly increased, possibly due to three equine ergonomics sessions (equine dentistry, orthopedics, internal medicine). An increase in self-efficacy and a decrease in internal locus of control may be associated with increased ability to perform risk adaptation measures but low perceived control over occupational morbidity or accidents. For example, participants perceived that they were more able to prevent dangerous situations by performing, e.g., restraining techniques, which indicates an increase in their self-efficacy. For the same reason, participants became more aware that some situations, e.g., a kick of a horse, may sometimes depend on external factors. In the context of veterinary medicine, it has to be noted that high levels of self-efficacy could also have detrimental effects on mental health, as in the case of high levels of empathy for the suffering of others [88]. However, this was not investigated in the present study but would be of interest in future studies taking into account the incidence of burnout and compassion fatigue in veterinarians and veterinary students [19].

### 4.10. Limitations

All our participants took part in the Active Microbreaks Program; therefore, the present study had no control group, and time–dose effects at MOS only partly mitigated this deficit of the study. Moreover, the self-selection of participants might have biased the results as students already experiencing MDP may have had a higher incentive to participate. Participants were not selected based on their chosen specialization. It might have been useful to create subsets with more similar physical demands. We did not control for exercises chosen, the number of repetitions of microbreaks, or the time spent performing the microbreaks. This is another limitation of the present study. Common and potentially limiting issues in self-reporting scales used in the present study are recall bias and social desirability. The choice of ergonomics topics was dictated by the veterinary staff of the university available to give them within the time frame allocated. For future studies, theriogenology should be included. As already mentioned, COVID-19 affected this study. Another limitation of this study was the use of new and therefore, as yet unvalidated items regarding LOC, SSE, and GSE, aimed explicitly at veterinary students. Acknowledging these limitations, the study presents only preliminary findings. For future research, the validity of the scales should ideally be investigated more extensively and in a larger group of students.

## 5. Conclusions

In this preliminary study, we found that veterinary students frequently suffered from MDP, potentially contributing to impairments later in their professional careers. Additionally, mental health issues pose a threat to the well-being of veterinary students. Based on the results of the present study, a program comprising microbreaks as well as ergonomics and safety education may be developed aimed at reducing MDP while improving self-efficacy and internal locus of control. It would ideally be possible to integrate an Active Phase of just 6 weeks into the veterinary curriculum, as several significant beneficial effects were observed in the present study.

## Figures and Tables

**Table 1:** Results of the Wilcoxon signed-rank test (Wilcoxon SOS–EOS) for Coping with Pain Strategy, sums of body regions with musculoskeletal discomfort, and pain (MDP) of the Standardized Nordic Musculoskeletal Questionnaire (NMQ), and scores of the STarT-Back Tool: At the time points in the week before the intervention started (SOS) and after the intervention after 12 weeks (EOS). The Median (Mdn) as well as Minimum to Maximum (Min–Max) are presented. The effect size after Cohen (r) is presented for the significant difference, *p* < 0.05. The long version of the items is presented in the footnotes. *n* = the total number of participants.

(*n* = 36)	SOS	EOS	Wilcoxon SOS–EOS		
	Mdn (Min–Max)	Mdn (Min–Max)	Z	*p* (2-Tailed)	r
Coping with Pain Strategy					
I do something (physical) ^c^	3.00 (1–5)	4.00 (0–5)	−1.280 ^a^	0.201	
I take something (medical) ^d^	1.00 (0–5)	2.00 (0–4)	−0.042 ^a^	0.966	
NMQ					
Sum MDP 12 months	4.00 (0–8)	3.00 (0–8)	−1.438 ^b^	0.151	
Sum Disability 12 months	0.00 (0–4)	0.00 (0–4)	−1.754 ^b^	0.079	
Sum MDP 7 days	2.00 (0–7)	1.00 (0–6)	−2.001 ^b^	0.045	0.33
STarT-Back Tool					
Total score	1.00 (0–6)	1.00 (0–8)	−0.232 ^b^	0.817	
Psychosocial subscale score	0.00 (0–5)	0.00 (0–5)	−0.182 ^a^	0.856	

^a^. Based on negative ranks. ^b^. Based on positive ranks. ^c^. When I have pain, I do something about it (e.g., exercise, relaxation, physiotherapy). ^d^. When I have pain, I take something for it (e.g., pain-relieving substances).

**Table 2:** Results of the Wilcoxon signed-rank test (Wilcoxon SOS–EOS) for the locus of control (LOC), specific self-efficacy (SSE), and general self-efficacy (GSE): At the time points in the week before the intervention started (SOS) and after the intervention after 12 weeks (EOS). The Median (Mdn) as well as Minimum to Maximum (Min–Max) are presented. The effect size (r) is presented for significant differences, *p* < 0.05. The long version of the items is presented in the footnotes. *n* = the total number of participants.

(*n* = 36)	SOS	EOS	Wilcoxon SOS–EOS		
	Mdn (Min–Max)	Mdn (Min–Max)	Z	*p* (2-Tailed)	r
Locus of control (LOC)					
Bad grade ^c^	1.00 (0–5)	2.00 (0–4)	−1.091 ^a^	0.275	
Horse kick ^d^	4.00 (2–5)	4.00 (1–5)	−1.236 ^b^	0.216	
Dog bite ^e^	2.00 (0–5)	3.00 (1–4)	−2.168 ^a^	0.030	0.37
Physical well-being ^f^	1.00 (0–4)	1.00 (0–4)	−1.613 ^b^	0.107	
Mental well-being ^g^	4.00 (2–5)	4.00 (3–5)	−0.426 ^a^	0.670	
Stay healthy ^h^	5.00 (3–5)	4.00 (3–5)	−0.884 ^b^	0.377	
Influence on safety ^i^	3.00 (1–5)	3.00 (1–5)	−0.765 ^a^	0.444	
Specific self-efficacy (SSE)					
What to do in pain ^j^	2.00 (0–5)	3.00 (1–5)	−4.104 ^a^	0.000	0.68
Prevent risky situations ^k^	3.00 (1–5)	4.00 (2–5)	−2.309 ^a^	0.021	0.38
Handle dangerous situations ^l^	3.00 (1–5)	4.00 (2–5)	−3.132 ^a^	0.002	0.52
Stay calm ^m^	3.00 (0–5)	4.00 (1–5)	−1.872 ^a^	0.061	
Regain health after injury ^n^	5.00 (2–5)	5.00 (2–5)	−0.215 ^b^	0.830	
Keep engagement ^o^	4.50 (2–5)	4.00 (1–5)	−1.076 ^b^	0.282	
General self-efficacy (GSE)					
Protect myself ^p^	4.00 (1–5)	4.00 (2–5)	−2.512 ^a^	0.012	0.42
Maintain physical well-being ^q^	4.00 (0–5)	4.00 (2–5)	−2.351 ^a^	0.019	0.39
Confident handling horses ^r^	4.00 (0–5)	3.00 (1–5)	−2.000 ^a^	0.046	0.33
Confident handling dogs ^s^	4.00 (2–5)	4.00 (1–5)	−0.166 ^b^	0.868	
Confident handling cats ^t^	3.00 (0–5)	4.00 (0–5)	−1.577 ^a^	0.115	

^a^. Based on negative ranks. ^b^. Based on positive ranks. ^c^. If I fail an exam, it was just bad luck. ^d^. If I take the right actions, I can avoid being kicked by a horse. ^e^. If I get bitten by a dog, it’s my own fault. ^f^. I only have a little influence on my physical well-being during my work. ^g^. I am the one shaping my mental well-being during my career as a veterinarian. ^h^. If I take good care of myself, I can stay largely healthy during my career as a veterinarian. ^i^. Other people have a big influence on whether I get into a risky situation with animals during my work. ^j^. I know what to do in situations that are often painful for me (during my work with animals). ^k^. I know how to prevent risky situations with animals (during my work). ^l^. I know how to behave in situations with animals that are likely to be dangerous (during my work). ^m^. I stay calm in risky situations with animals (during my work). ^n^. In the profession of veterinary medicine, it is possible to be injured by animals while working (human–animal interaction): Given that, I am still confident to get well again during my career as a veterinarian. ^o^. As a veterinarian, I can get into situations that I find emotionally demanding and/or traumatic: Given that, I am nevertheless confident of regaining my motivation and commitment during my career as a veterinarian. ^p^. I am confident that I know how to protect myself. ^q^. I am confident that I can maintain my physical well-being. ^r^. I am confident in handling horses. ^s^. I am confident in handling dogs. ^t^. I am confident in handling cats.

**Table 3:** Time–dose effect of weeks of the Active Phase: Significant Spearman’s rank correlation r_s_ (*p* < 0.05) of the Active Phase with responses to the questionnaires at the time point in the middle of the study after 6 weeks (MOS). Two items, as well as the sum of body regions with musculoskeletal discomfort and pain (MDP) in the preceding 12 months of the Standardized Nordic Musculoskeletal Questionnaire (NMQ), were significant. The long version of the items is presented in the footnotes. *n* = the total number of participants.

MOS (*n* = 36)	Spearman’s Rank Correlation r_s_	*p* (1-Tailed)
NMQ		
MDP hip 12 months ^a^	0.307	0.034
MDP knee 12 months ^b^	0.305	0.035
Sum MDP 12 months	0.355	0.017
Locus of control (LOC)		
Horse kick ^c^	−0.362	0.015
Specific self-efficacy (SSE)		
Regain health after injury ^d^	−0.365	0.014

^a^. Have you, at any time in the last 12 months, had trouble (ache, pain, discomfort) in one (or both) hip(s) and/or thigh(s)? ^b^. Have you, at any time in the last 12 months, had trouble (ache, pain, discomfort) in one (or both) knee(s)? ^c^. If I take the right actions, I can avoid being kicked by a horse. ^d^. In the profession of veterinary medicine, it is possible to be injured by animals while working (human-animal interaction): Given that, I am still confident of getting well again during my career as a veterinarian.
